# Production of eco composites based on natural rubber and recycled sugarcane bagasse waste to be utilised as a type of food contact

**DOI:** 10.1038/s41598-024-73296-w

**Published:** 2024-10-03

**Authors:** Nagwa A. kamel, E. S. Shafik, Y. M. Nabil, Salwa L. Abd El Messeih

**Affiliations:** 1 Microwave Physics and Dielectrics Department, Physics Research Institute, National Research Centre, Giza, Egypt; 2 Polymers and Pigments Department, Chemical Industrial Research Institute, National Research Centre, Giza, Egypt; 3 Central Laboratory of Residue Analysis of Pesticides and Heavy Metals in Food (QCAP), Agricultural Research Center (ARC), Cairo, Egypt

**Keywords:** Natural rubber, Sugar cane bagasse, Polymer composites, Physical properties, Food contact, Biophysics, Materials science

## Abstract

Natural fibres are abundant, renewable, and biodegradable, which has inspired numerous academics worldwide to investigate their possible applications in various industrial fields. The food packaging sector is seeking bio-based and biodegradable substitutes to increase sustainability. In this study, new composites were prepared from natural rubber (NR) and sugarcane bagasse fibres (SCB) with different concentrations of SCB (0, 2.5, 5, 10 &20 phr). The effect of SCB on the properties of natural rubber was studied before and after the alkaline treatment of the fibres. The biocomposites are characterized using Fourier transmission infrared spectroscopy, thermogravimetric analysis, scanning electron microscope, transmission electron microscope, and dielectric measurements in addition to rheological and mechanical analysis. The overall migration test for biocomposites loaded with 20phr SCB was performed to assess the biocomposite’s safety as food contact materials. The study’s results indicated that, adding SCB improved the conductivity, tensile strength, and elongation at break of natural rubber. Alkaline treatment strengthened the bonding between the filler and matrix and improved biocomposites’ thermal dielectric and mechanical properties. The overall migration test indicated that the alkaline treatment increased the overall migration to simulants. Accordingly, alkaline-treated NR-SCB biocomposites are effective eco-friendly food packaging candidates for certain types of food such as aqueous non-acidic products.

## Introduction

The development of alternate methods of using our natural resources has received much-needed force in recent decades as environmental protection has grown in importance as a global priority^1–4^. Natural fibres (NFs) when used as filler in composite materials provide various benefits, including increased mechanical performance, environmental friendliness, cost savings, and bio degradation^5^. In addition, recycling of agricultural waste will reduce agricultural waste accumulation problems^4,6^.

Since ancient times, natural rubber (cis-1,4-polyisoprene) has been used as a natural biopolymer. Numerous rubber products were discovered to use a relatively broad range of rubber types. Tires, mats, erasers, gloves, catheters and bandages are some examples of such products. Temperature resistance is one of the crucial factors in choosing a rubber for a particular end-use application, though other characteristics like chemical inertness, the physical characteristics of the finished product (such as tensile strength, resistance, and abrasion), and the kinds of fillers and additives that must be added to it to achieve the desired final properties are also taken into account. Natural rubber is one of the significant rubber types utilized in the food sector^7^. Nooun, Phatchariya, et al. prepared packing foam sheets from natural rubber and rice starch. The biocomposites filled with activated carbon aiming to absorb the ethylene gas that released from banana packaging. The results indicated that the biocomposite containing 15 phr activated carbon decreased ethylene levels and delayed fruit ripening and degradation^8^. Biocomposites from poly lactic acid (PLA) and cocoa bean shells(CBS) have been studied by Papadopoulou et al., it is found that, cocoa bean shells powder added antioxidant activity to the composite and enhance the swelling and barrier properties which render the PLA/CBS composites an extremely encouraging substance for active food packaging^9^. Pant, Ruby, et al., developed innovative packaging materials by reinforcing composite materials with natural fibres including jute, coir, and aloe vera. The data collected indicate that Jute material performs better than other materials that could be used in place of packaging material^10^. In order to create biocomposites that can also be utilised in hard packaging, coir fibres can be employed as a reinforcing fibre in combination with biodegradable polymers or plastics. Therefore, biodegradable and biopolymers which rely on naturally occurring materials like coir fibers can be thought of as the eco-friendly food packaging of the future^11^.

After the juice from sugarcane is extracted, the crushed cane (known as bagasse). It is one of the overabundant biomass residues provided by sugar industry in immense quantities.This waste is mainly consisting of cellulose, lignin, hemicellulose, ash, and wax^12^. They usually thrown away. According to a survey of the literature, SCB is preferred for producing high-quality green products because to its low production costs. This is mostly attributable to the sugar processing plants’ large supply of raw materials and their affordable pre-treatment costs^13^. The bagasse waste is used in some applications such as paper industries, feedstock, or biofuel^14^. Bagasse based composites offering potency as a core material substituting expensive, high density wood fibre board^15^. Effect of sugarcane bagasse on the mechanical and thermal characteristics of composites made of cassava starch and beeswax was discussed by Jumaidin, Ridhwan, et al., the study revealed that, the addition of the fibres improved the flexural, tensile, and impact strength^16^. According to research on Polypropylene composites reinforced with sugarcane bagasse fibres that had been subjected to alkali treatment (NaOH), materials made with untreated fibres had lower stiffness and strength^17^ .

Numerous short natural fibres have been added to natural rubber; however, the results were subpar compared to silica and carbon black reinforcements. Poor interfacial adhesion caused by incompatibility between the rubber substrate and reinforcement was mostly responsible for this. It has been demonstrated that surface modifications with bonding agents, alkali treatments, acetylation, and mercerization can improve inter-facial adhesion and, as a result, the mechanical properties of fibre-reinforced rubber composites^18^. In another study, chemically modified sugarcane bagasse fibres were used as a filler for cardanol resin. The results indicated improvement in the tensile and flexural strength and the thermal stability of the composite by increasing the bagasse fibres up to 15 wt%^19^.

Santos et al. used the ash from burning sugarcane bagasse (residue) as a source of silica to reinforce natural rubber. The study revealed improvement in the mechanical properties of the rubber^20^ .

This study aims to produce new green composites based on natural rubber and sugarcane bagasse waste (untreated and alkaline treated) as recycled renewable bio-fillers. The biocomposites’ dielectric, structural, mechanical, and thermal properties were studied. The overall migration test was carried out to examine the safety of the biocomposites for the intended use as food contact material.

## Materials and methods

Natural Rubber (NR) in the form of Smoked Malaysian Rubber (SMR), specifically SMR 20, was employed. Key properties include low dirt content (0.20% max), ash content (1.00%max), Nitrogen (N_2_) content (0.60%max), and volatile matter (0.80%max). It exhibited a minimum Wallace plasticity of 30 and a plasticity retention index of at least 40, serving as a vital material component in our study. Sugarcane fibres were collected from juice shops as waste material. Commercial-grade chemicals were utilized without further purification, including elemental sulfur (a curing agent), 1,3-Diphenylguanidine (DPG), mercapto-benzothiazole (MBT), stearic acid, and zinc oxide (ZnO) as activators, paraffin oil as a plasticizer, N-Cyclohexyl-2-benzothiazole sulphenamide (CBS) as accelerators, and 2,2,4-trimethyl-1,2- dihydroquinoline (TMQ) as antioxidants. Sodium hydroxide (NaOH) from alpha chemika.

### Preparation of the sugarcane bagasse (SCB) fibres

Sugarcane waste fibres are cut into small pieces (about 5 cm) and washed with water to remove the dust. After being left for three days under the sun, the sugarcane bagasse was ground into a fine powder and sieved using the USA-standard testing sieve No. 120 to achieve uniform particle size.

### Alkaline treatment of SCB (mercerization)

One of the oldest and most used chemical treatments for natural fibres is the alkali treatment. This procedure involves applying sodium hydroxide (NaOH) to the fibres to totally remove pectin, wax, oils, and other organic substances, as well as lignin and hemicellulose. In this work, 50 cm long bagasse fibres were soaked in a 10:1 liquor-to-water solution at 30 °C. The fibres were immersed for 5 h in the solution and then neutralized with diluted acetic acid. Then, the fibres were washed with distilled water many times to remove any NaOH that had adhered to the surface. A pH of 7 was kept throw-out. The fibres were then dried for 48 h at room temperature, ground into a powder, sieved using USA standard testing sieve No. 120 and used as bio fillers into the NR matrix^21^.

### Preparation, rheological characteristics and vulcanization of NR rubber composites

In accordance with ASTM D3182-21a^22^, natural rubber (NR) was combined with other compounded ingredients and curatives and placed onto an open two-roll mill with a 170-mm diameter, a 300-mm working distance, and a 24-rpm slow roll speed at a 1:1.25 gear ratio. Table 1 displays the basic formulation for NR rubber compositions.

As per ASTM D2084-19a, the rubber mixtures’ rheological properties were evaluated at 152 ± 1 °C using a moving dierheometer, MDR-one rheometer (TA Instrument)^23^.

The maximum torque (M_H_), minimum torque (M_L_), scorch time (ts_2_), optimum cure time (tc_90_), and cure rate index (CRI) were ascertained. Next, using a hydraulic press and a clean, polished mold measuring 15 cm × 15 cm × 0.2 cm, the NR composites were vulcanized at their optimal cure times (tc_90_) at a pressure of 4 MPa and 152 ± 1 °C. The following formula was used to determine the cure rate index:

1 $$\:\text{C}\text{u}\text{r}\text{e}\:\text{r}\text{a}\text{t}\text{e}\:\text{i}\text{n}\text{d}\text{e}\text{x}\:\left(\text{C}\text{R}\text{I}\right)=\frac{100}{\text{T}\text{c}90-\text{t}\text{s}2}$$

**Table 1:** Formulation of natural rubber biocomposites.

Ingredients, phr	Samples	
	NR-SCB Untreated	NR-SCB Treated
Natural rubber	100	100
Sugar cane waste	0, 2.5, 5, 10, 20	–
Treated sugar cane waste	–	2.5, 5, 10, 20
ZnO	4	4
Stearic acid	2	2
Paraffin oil	2	2
Sulfur	2.2	2.2
MBT	1	1
CBS	0.5	0.5
TMQ	1	1

### Fourier-transform infrared (FTIR)

Bruker VERTEX 80 (Germany) was used to measure FTIR in conjunction with Platinium Diamond ATR, which consists of a diamond disc as an internal reflector in the range 4000–400 cm^−1^ with a resolution of 4 cm^− 1^ and a refractive index of 2.4.

### Transmission electron microscope (TEM)

The particle size of the grounded bagasse fibres was measured using an accelerating voltage of 200 kV and the JEM-HR 2100 type of transmission electron microscope.

### Scanning electron microscope (SEM)

The polymer surface was studied by SEM/EDX, Philips XL30, Japan, with an accelerating voltage of 30 kV. at magnification 6000.

### Thermogravimetric analysis (TGA)

A computerized 7 series USA Perkin Elmer, thermal analysis equipment, was utilized to assess the thermal stability of the samples. Samples were stored in an air environment and examined across a 50–600 °C temperature range at a steady heating rate of 10 °C/min. Powdered aluminium oxide was utilised as a reference.

### Dielectric measurements

Using the Novocontrol Alpha Analyzer (GmbH concept 40, tan d[10^− 4^]), the permittivity (ε′), dielectric loss (ε″), and dc electrical conductivity (σ) of the prepared samples were measured in the frequency range of 10^1^ to 10^7^ Hz at 30^o^C. The resistance R, loss tangent tan δ, and capacitance c that were taken straight from the bridge served as the foundation for the computations^24^.

### Physico-mechanical properties of NR vulcanizates

Sheets of natural rubber Using an ASTM cutter, vulcanizates were formed into dumbbell-shaped specimens 5 cm long and 0.4 cm broad. A standard thickness gauge was used to measure the thickness of each specimen. A Zwick/Roell Z010 tensile tester machine with a load cell (Type: X force P and Nominal force: 10 KN) was used to determine the physico-mechanical properties (tensile strength, MPa, elongation at break, %, and hardness shore A) of the rubber composite vulcanizates both before and after accelerated thermal aging in accordance with ASTM D412-16 (2021)^25^. For the average, the physico-mechanical data were measured in five replicates.

### Overall migration test

The term “overall migration” describes the entire amount of non-volatile substance transported from material in contact with food to a certain equivalent solvent at a particular temperature and time. Often, the unit of measurement is expressed as milligrams (mg/kg) or milligrams (mg/dm^2^) of non-volatile substances per square decimeter of contact area or kilogram of food simulators.

Overall migration tests were performed in accordance with EN 1186 Migration Testing for Food Contact Materials. Using a borosilicate glass tube capped with a screw cap and internally laminated with Teflon, specimens with a surface area of 1 dm^2^ (10 cm × 10 cm and a thickness of 0.10 mm) were placed in contact with 100 mL of a preconditioned simulant at 40 °C, named as ethanol 10%, ethanol 95%, isooctane, and citric acid 3%. A surface/volume ratio of 10 dm^2^/L was attained. The usual EU surface/volume ratio of 6 dm^2^/kg food (6 dm^2^/L simulant) was used to compute the overall migration results. A weighted quartz capsule was filled with a known aliquot of the simulant from the contact solution, which was then evaporated to dryness until the weight remained constant. based on the variations in the weights. Five samples were used to average the results.

The overall migration study was conducted by the Food Contact Materials (FCMs) division of the Central Lab of Residue Analysis of Pesticides and Heavy Metals in Food at the Agricultural Research Center. The center has been accredited by the Finnish Accreditation Service (FINAS) since 2006, in accordance with ISO/IEC 17025:2017.

## Results and discussion

### Transmission electron microscope (TEM)

**Fig. 1:**
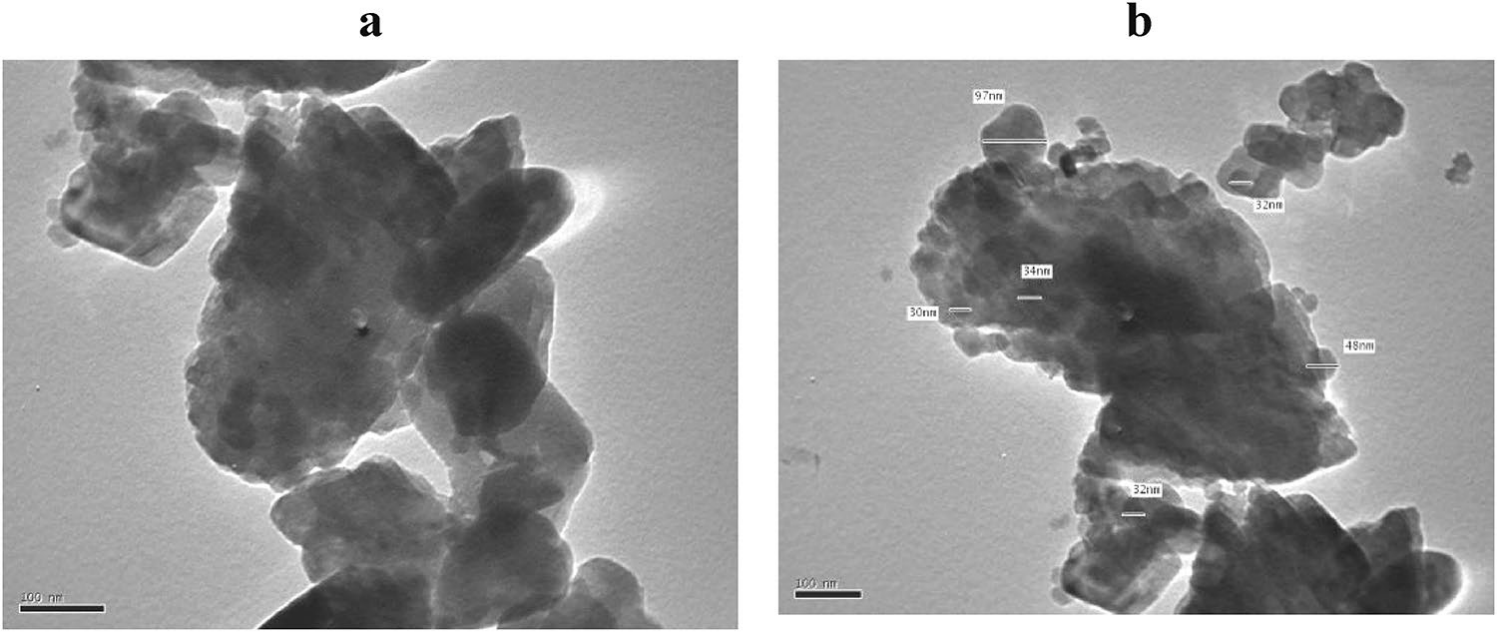
TEM micrographs for SCB powder (**a**) untreated (**b**) treated.

The TEM images of SCB powder before and after alkaline treatment are shown in Fig. 1. These figures indicate that the particles’ average size is about 50–70 nm in the case of untreated powder (Fig. 1a). After treatment, the average size was reduced to 30–48 nm(1b).

### Fourier transform infrared spectroscope (FTIR)

**Fig. 2:**
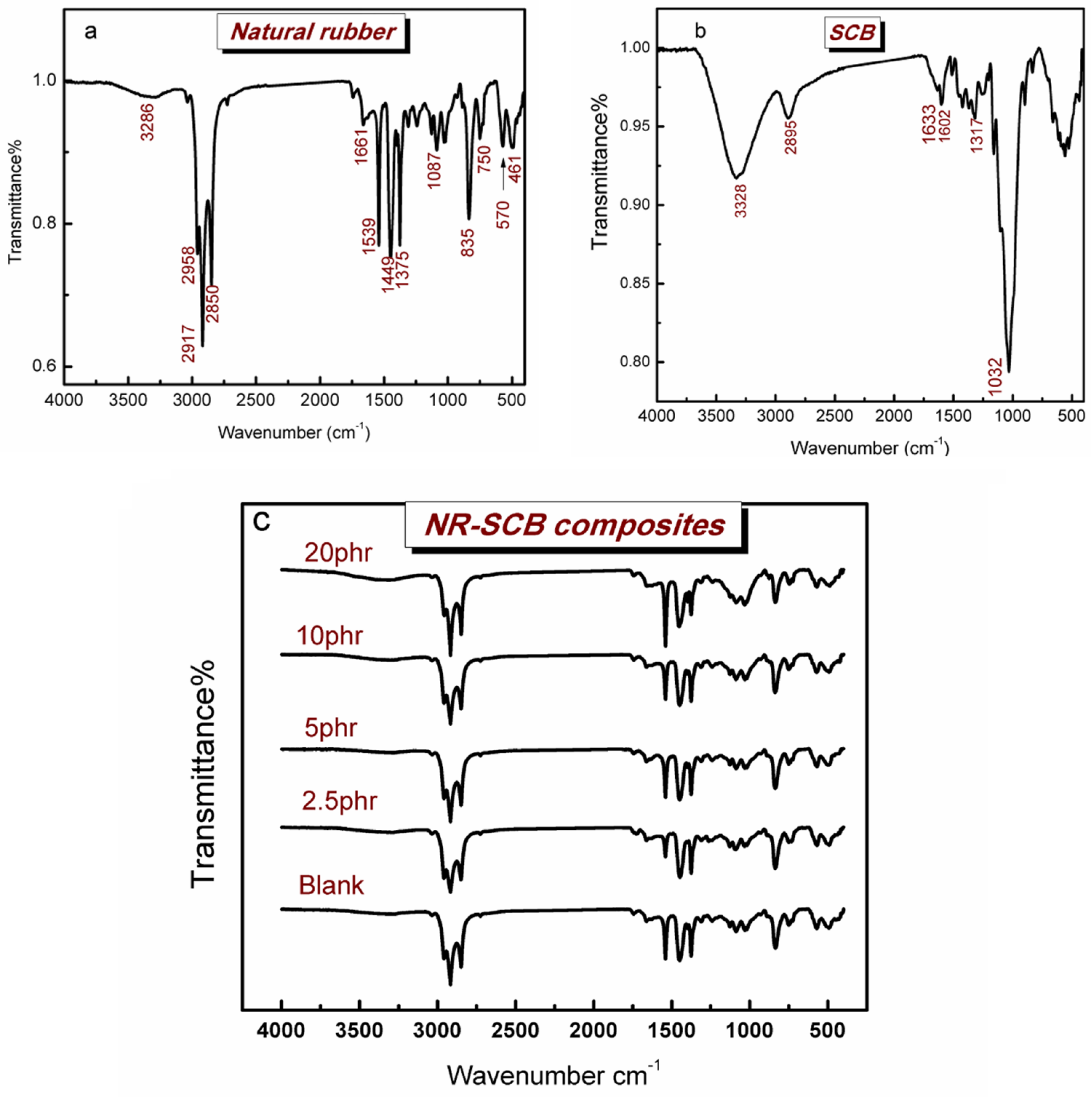
FTIR spectra of (**a**) Natural rubber, (**b**) SCB filler, and (**c**) NR-SCB biocomposites with different concentrations of untreated bagasse.

Figure 2 indicates the FTIR spectra of natural rubber (NR), SCB powder, and NR-SCB composites with different concentrations of SCB. In the spectrum of natural rubber (NR) (Fig. 2a), the main functional groups were as follows: 3285 cm^-1^: O–H stretching vibration, The 3-shoulder peak appears from 2850 to 2958 cm^-1^ which represents the natural rubber backbone (2960 cm^-1^: CH_3_ Stretching vibration, 2917 cm^-1^: methylene group CH_2_ asymmetry stretching and 2850 cm^-1^: Methylene CH_2_ symmetrical stretching)^26^. 1661 cm^-1^: C = C Stretching vibration, 1530 cm^-1^: CH stretching and NH bending of amide II, 1449&1375 cm^-1^: CH_2_ and CH_3_ deformation, 835 cm^-1^: =CH wagging^27^.

Figure 2b stands for the FTIR spectrum of the row SCB. As seen from the spectrum, the major absorption bands of SCB appeared at 3328 cm^-1^corresponding to O–H of cellulose, 2895 cm^-1^ assigned for C–H stretching,1630 cm^− 1^ characteristic for O-H bending vibration of water that absorbed by the sample; 1603 cm^− 1^ assigned to aromatic rings’ C = C skeletal stretching vibration, indicating the presence of lignin in the bagasse^28^. The band around. 1259 cm^− 1^ was attributed to the stretching of acetyl groups in the hemi cellulose. and the strong band at 1032 cm^− 1^ refers to C-OH stretching of the cellulose structure^29^. The band at 897 cm ^− 1^ corresponded to CH rocking vibration of cellulose fibres^30^.

Figure 2c, Displays the spectra of NR based composites with different concentrations of SCB (untreated). The functional groups are similar in all bio- composites spectra, although, their intensities are changing among different concentrations. Furthermore, the band at 3285 cm^-1^ (O–H stretching vibration) increased in intensity up to 10hpr. In addition, the frequency of the band was shifted to higher wave number by increasing the SCB loading the spectra revealed possible hydrogen bonding between filler and NR matrix.

**Fig. 3:**
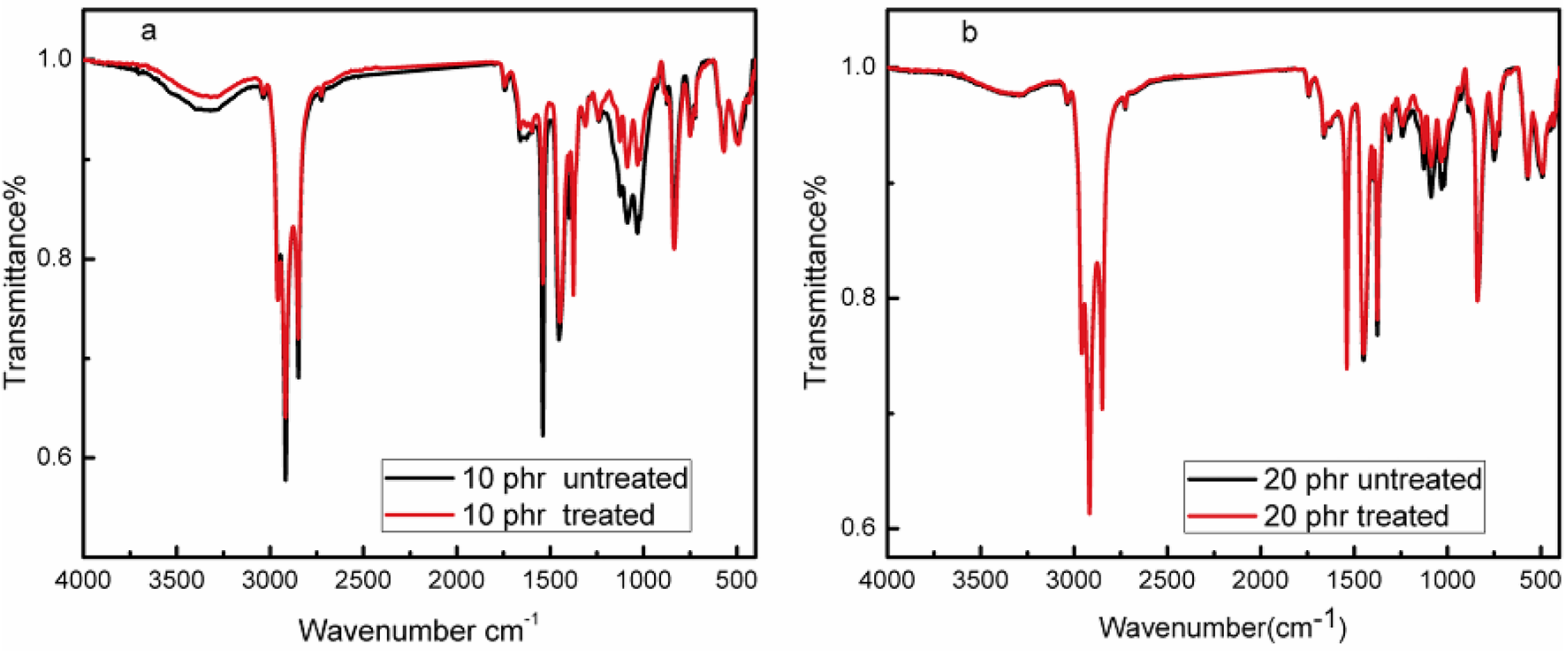
FTIR spectra of NR-SCB 10&20 phr biocomposites before and after treatment.

Figure 3(a & b) compared the spectra of the biocomposites incorporated with 10 &20 phr SCB for the purpose of conciseness prior to and following alkali treatment. As seen from the spectra, after alkaline treatment, the spectra revealed a decrease in the intensity of cellulose’s O-H stretching vibration at 3360 cm^− 1^. Otherwise, It has been observed a decrease in the intensities of most absorption peaks as 1125, 1086, 1032,1539, 1625, 1749, 1259, and 896 cm^− 1^ for the treated biocomposites compared to the untreated ones. The results suggest that the alkaline treatment eliminated the hemicellulose and lignin adhered to the fibre surface. Comparable results obtained by Prabhakar, M. N., et al. They studied peanut shell powder (PSP) as a natural reinforcer for epoxy resin.the results reported decrease in the intensities of some bands and attributed these decrease to the removal of impurities after alkaline treatment of the fibres^31^ The results also aligned with previous findings following alkaline treatments of natural fibres^32,33^.

### Dielectric and conductivity measurements

Another method for testing the biocomposite interface and examining how chemical treatment of the SCB fibres affects the composites’ inter-phases is dielectric spectroscopy technique.

**Fig. 4:**
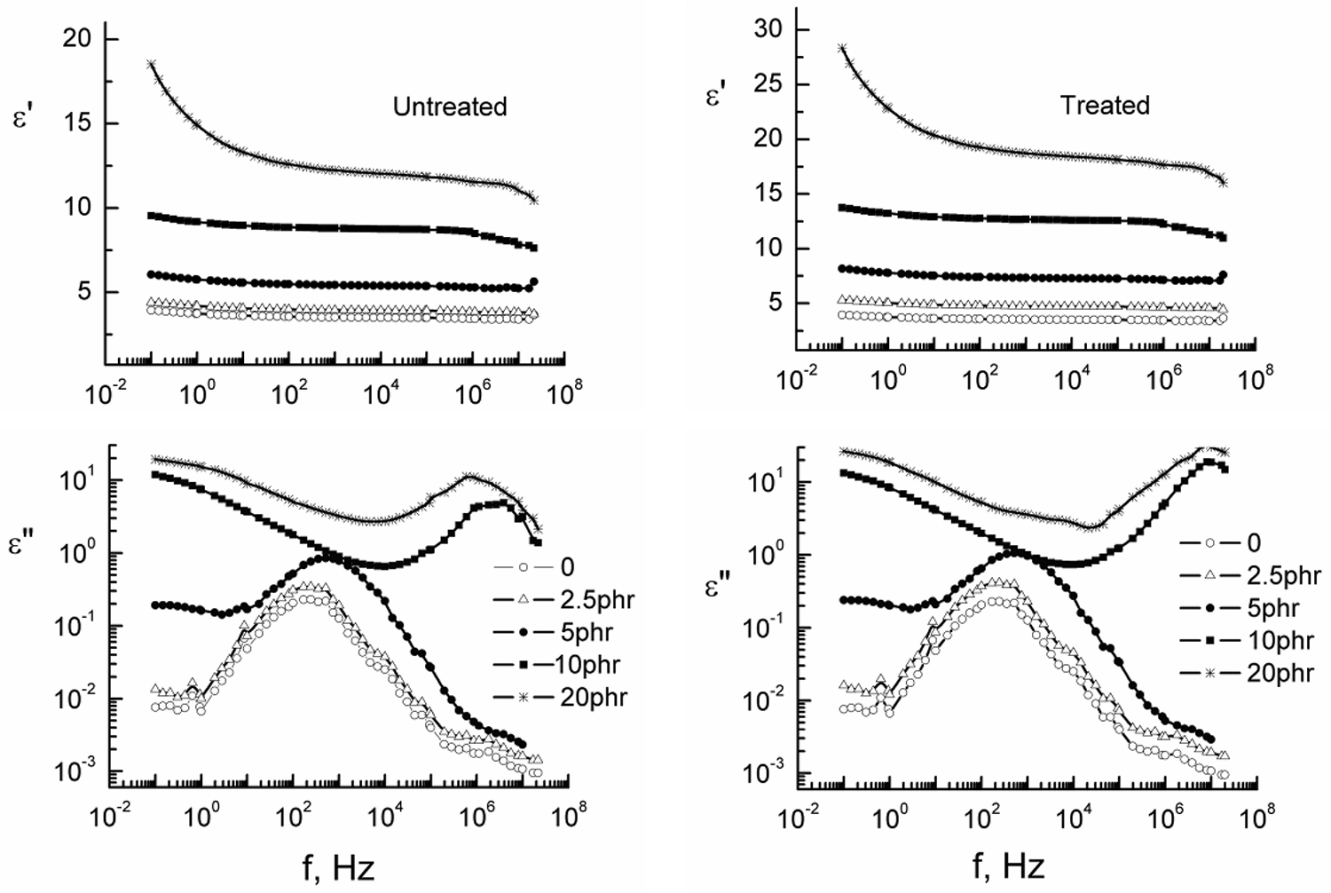
The permittivity ε′ and dielectric loss ε″ for untreated and alkaline treated NR-SCB biocomposites with different concentrations of SCB.

Both the dielectric parameters, permittivity ε′ and loss ε″ were given in Fig. 4 before and after treatment as a function of the frequency f. These figures reflect decrease in ε′ by increasing the applied f. The progressively declining in ε′ may be as a result of the dipoles of rubber molecules were unable to rotate parallel regarding the rapid periodic phase reversal of electric field which form time lag between the applied f of dipole and that of electric field^34,35^.

In this wealth, there is no either ion dispersal or charge accumulated. This finding was for samples loaded with concentrations up to 10 phr SCB but in the case of 20 phr, a sharp decrease in ε′ values was found before and after treatment. This may be explained as in the measured f range; the dielectric relaxation comprises the polarization due to the dipolar rotational, which depends upon the molecular organization of the material. At higher f values, the rotating movement of the molecules lags at the back of the electric field, showing a reduction in ε′ values with increasing frequency for all samples under test^26^.

In addition, the curves of ε” against the frequency f given in Fig. 4 are wide indicative of the fact that besides the electrical conductivity, various relaxation mechanisms could be present^36^.

**Fig. 5:**
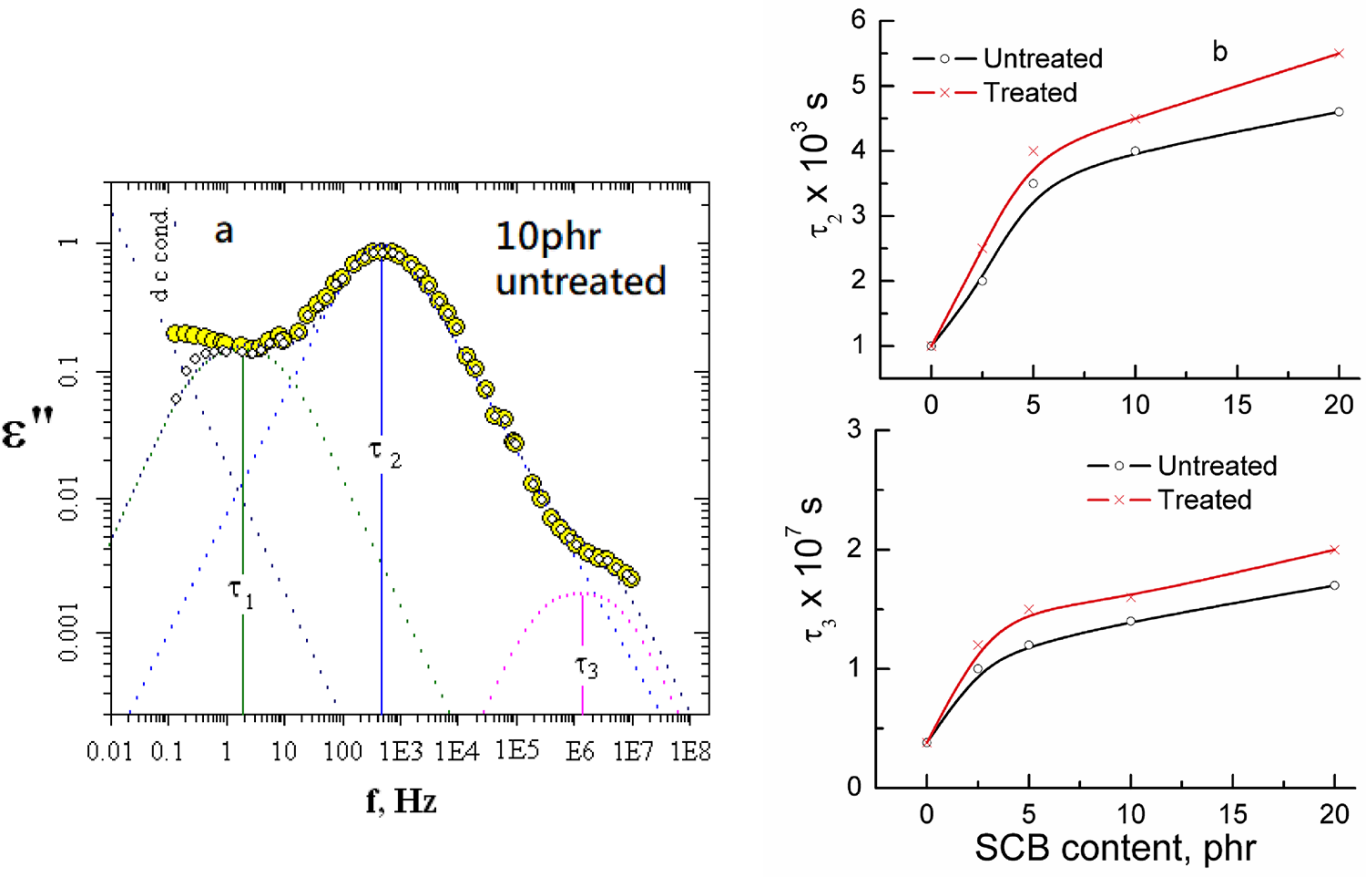
(**a**) Example of the analyses for 10 phr untreated, (**b**) The second τ_2_ and third τ_3_ relaxation times versus SCB content

The conductivity term was subtracted, and the absorption curves linking the applied frequency f to the dielectric loss ε” were then analysed in terms of the superposition of a Havriliak-Negami function and Fröhlich functions using the formulas previously provided^26^. An example of the analyses is given in Fig. 5a. The first relaxation appeared at a low-frequency range with relaxation time around 0.25–0.35 s could be ascribed to the combination of both the interfacial polarization and the electrode polarization is known as the Maxwell–Wagner–Sillars (MWS) effect^37,38^. This process results from the free components previously introduced to rubber becoming immobile in elements that increase the media’s conductivity, allowing charges to move in the applied electric field.

It is appealing to note that MWS relaxation is frequently perplexed with ionic conduction as these two phenomena are invented from charge carriers’ mobility. Previous research on molecular relaxation in an isotropic material, like acrylic polymer and hydroxyl propyl cellulose, revealed that ionic conductivity dominated the sample’s spectra at low frequencies^39^. It was encouraging to see that There was no increase in the relaxation time associated with this region, τ_1_, before or after treatment due to the filler content.

The second and third regions appear at a higher frequency range with relaxation time τ_2_ and τ_3_ and lie in the range of 10^− 3^–10^− 6^ s respectively, were fitted by Havriliak–Negami and Fröhlich functions could be due to the orientation of the large and small aggregates as a result of the main chain movement. The relaxation times associated with these regions were found to increase by increasing filler content. This increase reflects filler-polymer interaction rather than filler-filler interaction. This interaction increases the molar volume of the rotating units, which leads to an increase in both τ_2_ and τ_3_^39^. Both τ_2_ and τ_3_ before and after filler treatment were illustrated graphically in Fig. 5b. This figure reflects a pronounced increase in both τ_2_ and τ_3_ values by increasing the filler ratio up to 5phr, after which this increase becomes very small. This result was taken as further justification for the network formation via the systems under investigation.

**Fig. 6:**
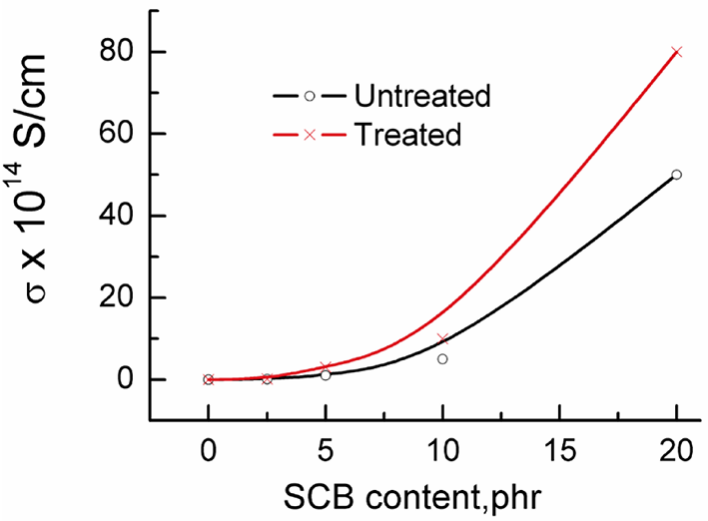
Electrical conductivity σ versus the SCB content before and after treatment.

The electrical conductivity was evaluated from the measured ac ones and the obtained results are given in Fig. 6. From the graph it is recognized that σ values increase by the filler increasing either treated or untreated that reflect an increase in the system polarity. The increase in σ was within the order of 10^14^ S/cm i.e., does not affect the insulation properties of the composite. This finding suggests the use of composites for insulation purposes^40^.

### Thermo gravimetric analysis (TGA)

**Fig. 7:**
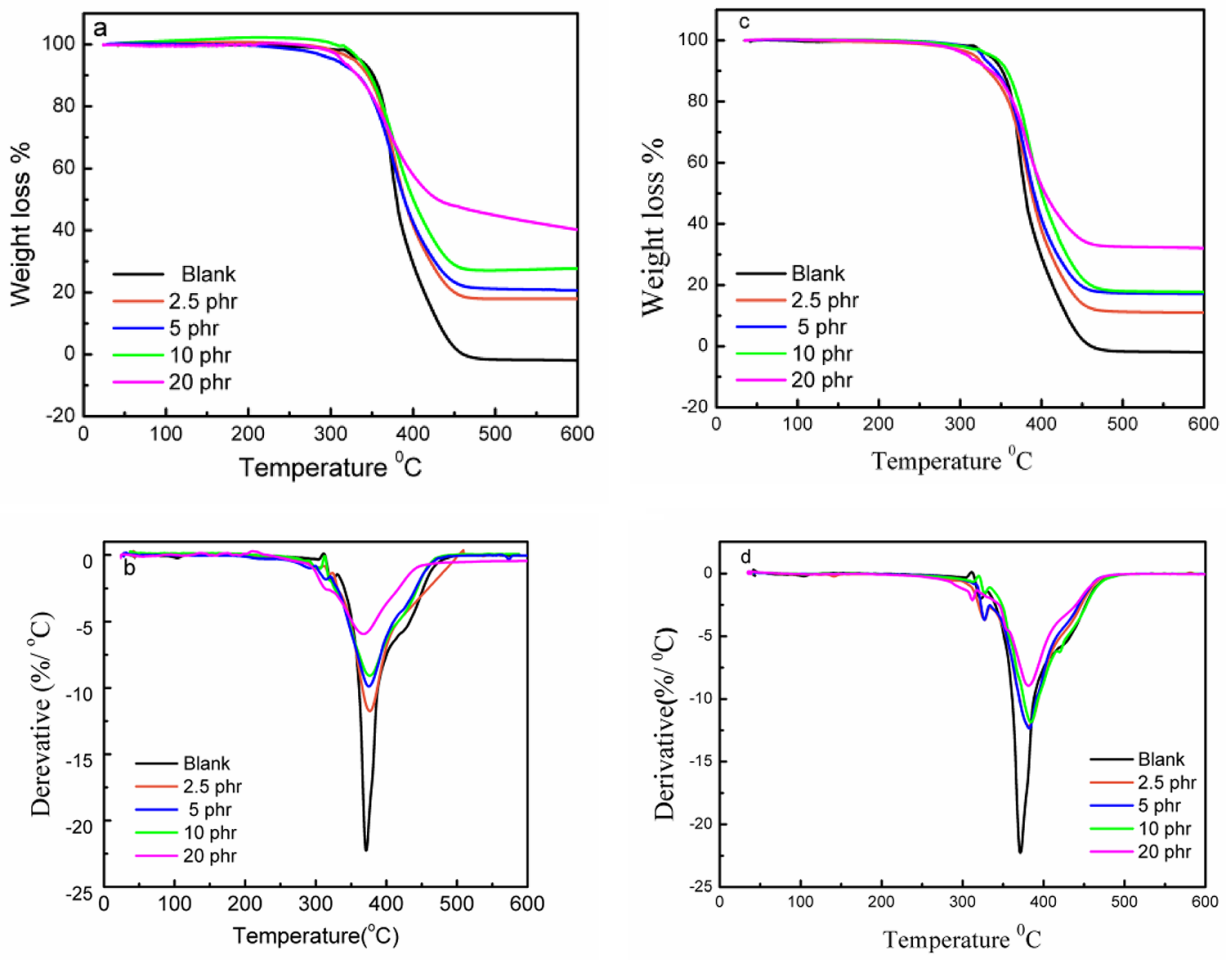
(**a, c**) TGA curves for NR-SCB biocomposites before and after alkaline treatment respectively, (**b, d**) derivative thermogravimetry (DTG) curves for NR-SCB biocomposites before and after alkaline treatment respectively (at heating rates of 10 °C/min).

The thermal stability of the NR-SCB biocomposites was examined by the thermo gravimetric analysis technique (TGA). Figure 7(a-d) represents the TGA profiles of the biocomposites with untreated and alkaline treated bagasse fibres, along with the derivative thermogravimetry (DTG) curves.

For natural rubber, the TGA curve characterized by a single decomposition process starts from 357.58 to 394.44^o^C. As shown, the incorporation of bagasse fillers did not alter the degradation attitude, i.e., all composites (treated and untreated) show single-process degradation. The degradation parameter T_deg_ and the residue at 600^o^C of the degradation process are estimated from the TGA and the derivative d m/ d T curves and tabulated in Table 2 to compare and identify the changes due to the reinforcement filler and due to the used fibre treatment.

As indicated from the results, an increase in the fibres ratio was accompanied by increasing in the residual content from − 1.5 for NR to 40.43% for 20wt% untreated SCB at 600^o^C. The remaining residue is attributed to the remaining lignin and inorganic tough materials, which need high temperatures to be totally decomposed^41^. Comparing the residual content of the composites with the untreated and treated SCB, it is noticed that the residual decreased for the alkaline treated samples than the untreated, which is a result of the lower content of hemicellulose, wax, and lignin due to the treatment process.

From Table 2, by incorporation of SCB, T_deg_ (the temperature at which maximum degradation took place), which is always used as an indicative parameter for the stability of rubber composites, increased about 4-5^o^C in NR-SCB composites by increasing the fibre ratio up to 10phr. Otherwise, the T_deg_ values increased after alkaline treatment. These results suggested that incorporating SCB filler up to 10phr and the alkaline treatment improve the thermal stability of NR-SCB biocomposites. The primary cause of the alkali-treated composites’ superior thermal stability over the untreated composites is their enhanced crystallinity following treatment.

Former studies confirmed the improvement of thermal stability of polymers by incorporation of SCB^16,19^.

**Table 2:** Thermal decomposition parameters for the biocomposites (NR-SCB) treated and untreated in terms of decomposition temperature (T_deg_) and mass residue at 600 ^o^C.

Composite with SCB content (phr)	Treatment	Decomposition Temperature (T_deg_) (°C)	Residual (%) at 600 ^0^C
0		371.24	-1.5
2.5	Untreated	376.32.5	15.4
	Treated	381.6	11.14
5	Untreated	376.2	20.5
	Treated	382.5	17.35
10	Untreated	375.18	27.44
	Treated	381.5	18.5
20	Untreated	367.2	40.43
	Treated	384.1	32.09

### Scanning electron microscope (SEM)

**Fig. 8:**
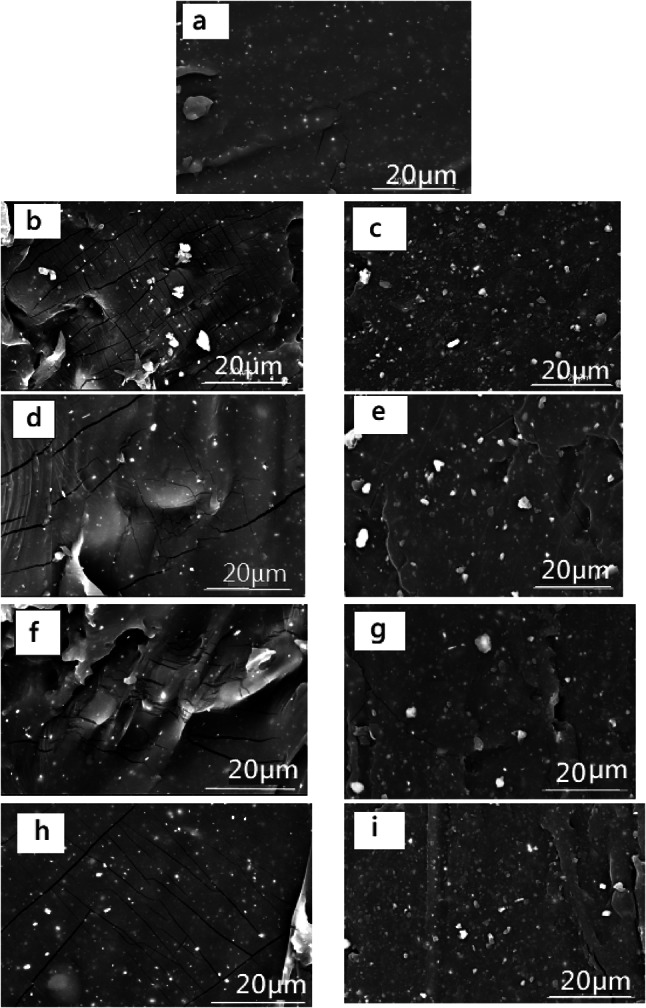
SEM images of NR-SCB biocomposites with different SCB content (**a**) Blank (NR) (**b**) 2.5 (**d**) 5 (**f**) 10, and (**h**) 20 phr SCB untreated (**c**) 2.5 (**e**) 5 (**g**) 10 (**i**) 20 phr treated biocomposites X6000.

Usually, the key factors affecting how filler and polymer interact are surface chemical structure of the filler, filler distribution, and surface area^42^. Figure 8. represents the SEM micrographs for NR, NR-SCB bio-composites with treated and untreated fibres. From the images, it is noticed that, pure natural rubber without filler (Fig. 8a) the surface of the samples was smooth with minor rankles. after loading the SCB filler, the surfaces became rough, and the surfaces contained cracks in all concentrations of SCB.

The bio-composites filled with alkaline-treated SCB filler [SEM images f-i], the distribution of the filler in the samples was more homogeneous. No or very few cracks were observed, even at high concentrations of the filler. Hence, SEM micrographs indicated compatibility enhancement between the polymeric matrix and the fibres. It is expected that the removal of lignin, hemicellulose, pectin, wax, and oils from the fibres will leave more cellulose molecules exposed at the surface, increasing the number of potential reaction sites and enhancing the attachment of fibres to the rubber matrix^43,44^.

### Rheological and mechanical analysis of NR-SCB biocomposites

Table 3 displays the rheological parameters of the produced NR composites, including minimum torque (M_L_), maximum torque (M_H_), torque difference (ΔM), scorch time (ts_2_), curing time (Tc_90_), and CRI (min^-1^).M_L_ is the unvulgarized elastomer’s stiffness and viscosity value; it is defined as the value that appears at the bottom of the torque-time diagram. In the curing test, the compound’s maximum torque (a measure of its stiffness) is recorded by MH. The term Tc_90_ refers to the duration needed to achieve 90% of the maximal torque. On the other hand, the time required to raise the minimum torque by two units is known as ts2. Moreover, the pace of the vulcanization process is measured by the curing rate index (CRI)^45^.

**Fig. 9:**
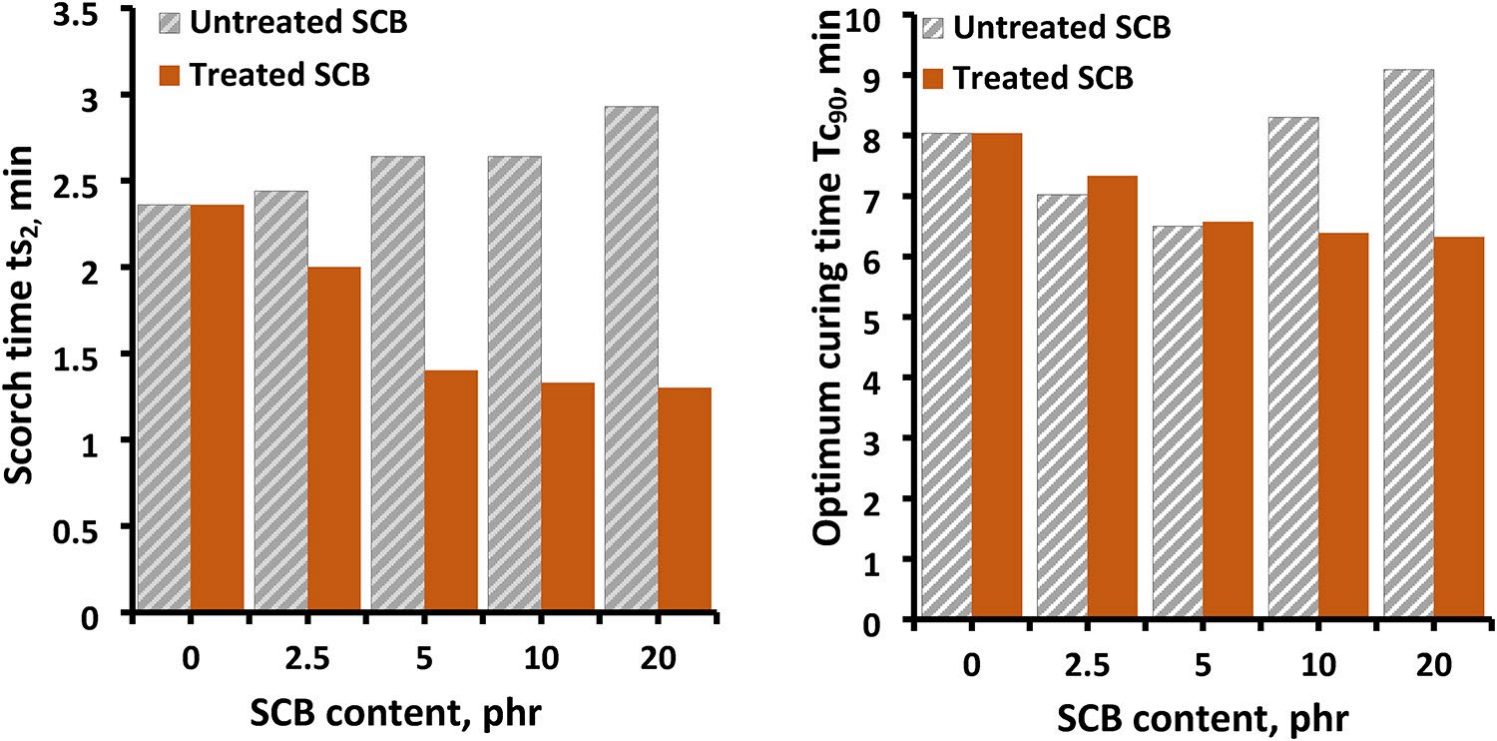
Scorch time and optimum curing time versus SCB filler content for NR biocomposites.

According to the data shown in the Table 3, it is evident that the M_H_ values for the NR-SCB and NR-SCB treated composites exhibit greater values compared to the blank NR. This finding suggests that the inclusion of sugar cane waste fibres resulted in a decrease in the mobility of the macromolecular chains of the NR, leading to an increase in the hardness and stiffness of the composite material^45,46^. Furthermore, an increase in both the maximum and minimum torques was seen in the rubber composite containing alkali-treated sugar cane fibre. This phenomenon may be explained by the alkali-treated natural fibre promoting the formation of new crosslinks and improved interactions between the fibre and rubber. The composite’s scorch time and curing time exhibited a steady reduction as the filler quantity increased. However, it was observed that the utilization of an alkali treated filler resulted in a reduction in the curing time of the composite in comparison to the untreated fibre filled composite as shown in Fig. 9. The dispersion of the filler in the rubber matrix was facilitated by the alkali treatment, which established molecular bridges at the interface between the filler surface and matrix.

**Table 3:** Rheometer characteristics for NR-SCB biocomposites.

Composite with SCB content (phr)	Rheometer characteristics					
	M_L_ d.NM	M_H_ d.NM	ΔM d.NM	ts_2_ (min)	Tc_90_ (min)	CRI (min^-1^)
**0**	1.30	8.40	7.10	2.36	8.04	17.30
**2.5**	1.23	8.06	6.83	2.44	7.02	21.83
**5**	1.16	8.16	7.00	2.64	6.50	25.91
**10**	1.14	8.36	7.22	2.64	8.30	17.66
**20**	1.13	9.13	8.00	2.93	9.09	16.23
**2.5 T**	1.30	8.60	7.36	2.00	7.33	18.76
**5 T**	1.23	8.91	7.68	1.40	6.57	19.34
**10 T**	1.30	9.13	7.83	1.33	6.39	19.76
**20 T**	1.37	9.66	8.29	1.30	6.32	19.92

### Physico-mechanical properties of NR-SCB biocomposites

**Fig. 10:**
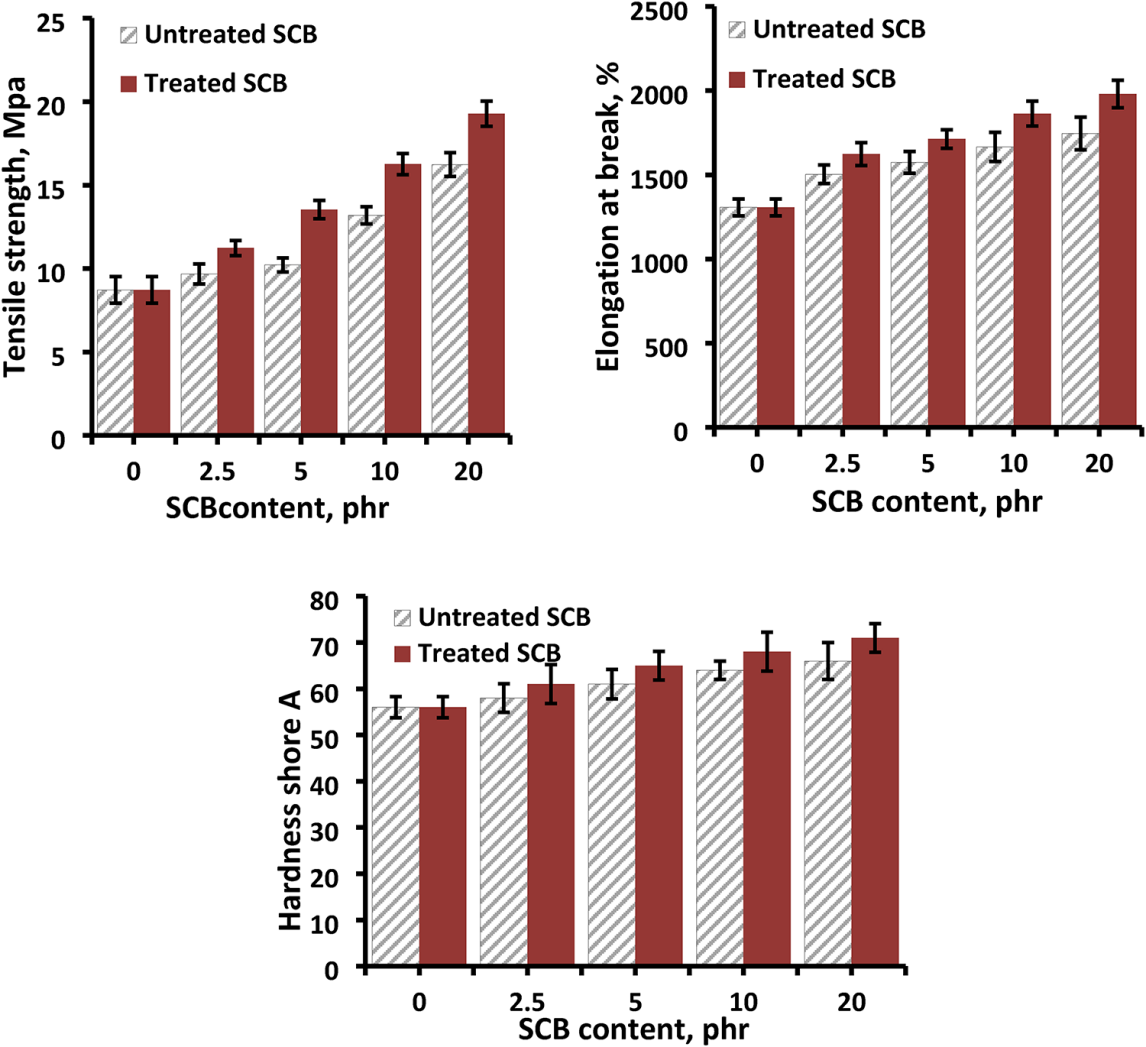
Mechanical properties versus SCB filler content for NR-SCB biocomposites.

The mechanical characteristics of natural rubber filled with untreated and alkali-treated sugar cane fibre are depicted in Fig. 10. The tensile strength exhibited a notable improvement, rising from 8.72 to 16.23 MPa in the case of untreated sugar cane waste fibre. While the tensile strength increased from 8.72 to 19.28 MPa in the case of alkali-treated sugar cane fibres. This behavior is consistent with several researchers’ reports^37,47^.

As shown in Fig. 10, the alkali treated sugar cane improved tensile strength by 13% compared with natural rubber filled with 20 phr of untreated sugar cane waste fibre. Also, Fig. 10 illustrates an improvement in both elongation at break and hardness shore A for natural rubber composites filled with alkali treated sugar cane waste when compared to untreated sugar cane.The improvement in mechanical properties of natural rubber filled with alkali-treated sugar cane due to alkali treatment is mostly used to remove several components from NFs, such as lignin, pectin, and hemicelluloses. First, alkali treatment can increase the total number of reaction sites by raising the concentration of cellulose on fibre surfaces. Furthermore, during alkali treatment, the amorphous sections of the fibres (lignin and hemi cellulose) are removed, resulting in materials with a higher crystallinity, which may cause the surface of NFs to become rougher.

Consequently, following the application of alkali treatment, there is a significant enhancement in the adhesion properties of NFs. This improvement may be attributed to the concurrent rise in available reaction sites and surface roughness^40^.

Comparable results were obtained by Khrongkhwan Yotkuna et al. which reported improved tensile strength and decrease elongation at break with increasing SCB content in rubber compounds^48^.The results also were consistent with previous researches which confirm that mechanical qualities including tensile strength (MPa), tensile modulus (MPa), flexural strength (MPa), and flexural modulus (MPa) are improved when sugarcane bagasse fibre is reinforced with various polymers^49^.

### Overall migration test

**Fig. 11:**
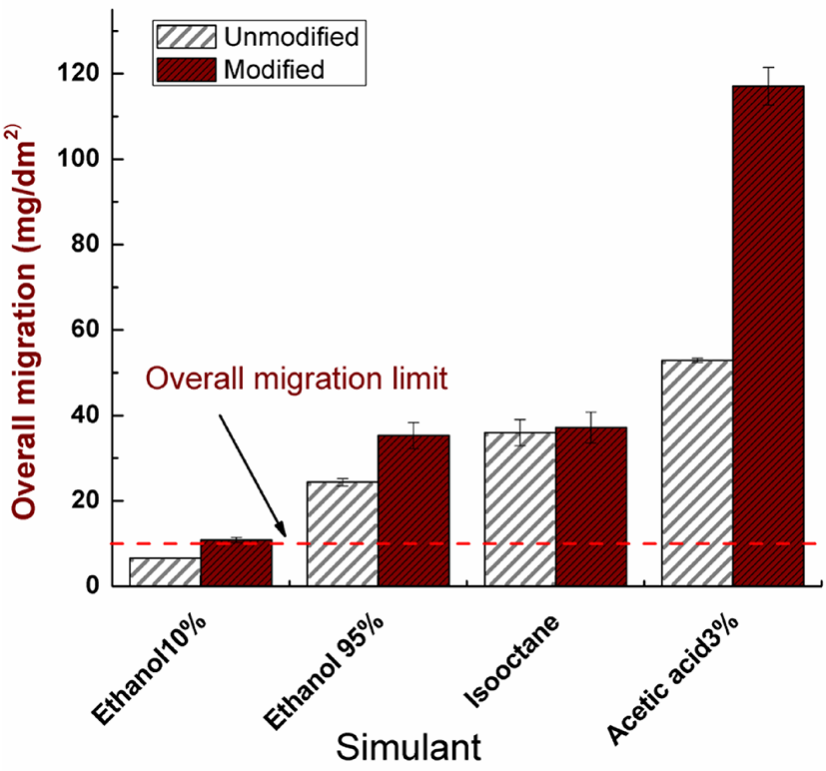
Overall migration versus simulants for untreated and treated biocomposites loaded with 20 phr SCB.

Although chemical modification methods have been effective in enhancing the fibre-matrix adhesion for several polymeric materials, it appears that no prior attempt has been made to explore the effect of these chemicals on food safety. In this study the selected composites (20phr NR-SCB) before and after filler modification were tested against 4 food simulants, specifically 10% (v/v) ethanol-water mixtures ethanol is a simulant for aquas food, which have hydrophilic nature, 3% acetic acid, a simulant for the acidic foods, Isooctane and 95% (v/v) ethanol are fatty food simulants which usually used to replace olive oil^50^. The data are presented in Table 4; Fig. 11.

**Table 4:** Overall migration of untreated and alkaline-treated natural rubber NR-SCB 20phr in 10% ethanol, 3% acetic acid, 95% ethanol, and isooctane before and after alkaline treatment. The contact conditions were 10 days at 40 °C.

Simulant	Overall migration ± SD (mg/dm^2^)	
	Untreated	Treated
Ethanol 10%	6.6 ± 0.046	10.86 ± 0.59
Acetic acid 3%	52.9 ± 3.07	117.1 ± 3.63
Ethanol 95%	24.4 ± 0.85	25.3 ± 3.04
Isooctane	35.96 ± 0.48	37.16 ± 4.42

Table 4 shows that the alkaline modification leads to an increase in the migration of chemicals to the simulants. This increase was pronounced in the case of acetic acid simulant. This may be due to the chemicals involved in the process. The values of ethanol 95%, Isooctane, and acetic acid simulants exceed the migration limits. higher migration levels were recorded for acetic acid for both modified and unmodified samples, and this is probably due to the acidic conditions, which may cause the lignocellulosic material (cellulose and lignin) of the SCB fibres to break down and leak out from the samples^51^.

The lowest value recorded was ethanol 10%, which was below the applicable migration limit of 10 mg/dm^2^ food in the EC 10/2011 regulation^52^ as expressed by the red dashed line in Fig. 11. Previous studies on natural fibres reinforced polymers show parallel results, Popa, E.E., et al., studied the migration in distelled water from polylactic acid containing cellulose fibres.The findings showed that the examined samples only went within the permitted^53^. Ethanol10 and distilled water are both simulants for aquas food.

The obtained results suggest using these composites in packaging of certain types of foods [aquas foods]. Moreover, the alkaline treatment is not the preferable technique when using the composites in packaging applications due to the chemicals involved.

## Conclusion

In this study, biocomposite materials based on natural rubber filled with sugar cane bagasse waste with different concentrations have been prepared. SCB fibres have been subjected to chemical treatment using sodium hydroxide10% solution. The biocomposites are characterized by different tools to investigate the effect of filler loading and alkaline treatment on the properties of the NR-SCB biocomposites. The results demonstrated that, inclusion of bagasse powder leads to increasing the thermal stability, conductivity and dielectric permittivity of the NR. FTIR and TEM confirmed the removal of the hemicellulose and lignin layer from the fibres surface. SEM images pointed to improved interaction between SCB-treated fibres and the rubber matrix compared to untreated fillers.An improvement in the elongation at break, hardness, the maximum and minimum torques was noticed by increasing filler ratio and for biocomposites filled with alkali-treated waste compared to untreated counterparts. The overall migration test results indicate increased migration to the simulants from the alkali-treated biocomposites, specifically in the acetic acid simulant. NR-loaded with treated 20phr SCB are safe to use in the packaging of aquas non acidic food. As overall conclusion, the achievement of producing composites with neat physical qualities that are superior to those of natural rubber utilising inexpensive and sustainable wastes and also analysis of the impact of alkaline treatment—which is often used to treat natural fibers—on food safety that is what makes this research innovative.

## Data Availability

All data generated or analysed during this study are included in this published article [and its supplementary information files].
